# Extreme Hyperthermia Due to Methamphetamine Toxicity Presenting As ST-Elevation Myocardial Infarction on EKG: A Case Report Written With ChatGPT Assistance

**DOI:** 10.7759/cureus.36101

**Published:** 2023-03-13

**Authors:** Jeffrey M Schussler, Cerin Tomson, Mark P Dresselhouse

**Affiliations:** 1 Cardiology, Baylor University Medical Center, Dallas, USA; 2 Cardiology, Baylor Scott & White Heart and Vascular Hospital, Dallas, USA; 3 Internal Medicine, Baylor University Medical Center, Dallas, USA; 4 Emergency Medicine, Baylor University Medical Center, Dallas, USA

**Keywords:** methamphetamine intoxication, st-segment elevation myocardial infarction (stemi), stemi, st-elevation myocardial infarction (stemi), methamphetamine, chatgpt

## Abstract

We present a case report of a 37-year-old male who presented to the emergency department with altered mental status and electrocardiographic changes suggestive of an ST-elevation myocardial infarction (STEMI). He was ultimately diagnosed with extreme hyperthermia, secondary to drug use, which was managed promptly with supportive measures resulting in a successful outcome. This case highlights the importance of considering drug-induced hyperthermia as a potential cause of altered mental status and EKG changes in patients, especially in those with a history of drug abuse.

## Introduction

We report a case of a 37-year-old man who presented to the emergency department with altered mental status and electrocardiographic changes suggestive of an ST-elevation myocardial infarction (STEMI). There was initial concern for a STEMI (based solely on EKG findings), but he was quickly diagnosed with extreme hyperthermia secondary to methamphetamine use. Successful management included prompt cooling measures, aggressive fluid resuscitation, and supportive care. This case underscores the importance of considering drug-induced hyperthermia as a potential cause of altered mental status and EKG changes, especially in patients with a history of drug abuse. Early recognition and treatment of hyperthermia are critical in order to prevent life-threatening complications and improve patient outcomes.

## Case presentation

A 37-year-old male with a past medical history of HIV, hypertension, polysubstance abuse (methamphetamine), and homelessness was found unresponsive in a parking lot and transported by emergency medical services to our emergency department. Upon arrival, he was noted to be hypoxic and tachycardic with a heart rate in the 180s. In the emergency department, he had a Glasgow Coma Scale score of 6 and a gaze deviation. His initial blood pressure was 80/40, and the core temperature was noted to be extremely elevated at 108.4°F (42.4°C), and he was promptly intubated due to hypoxia.

**Figure 1:**
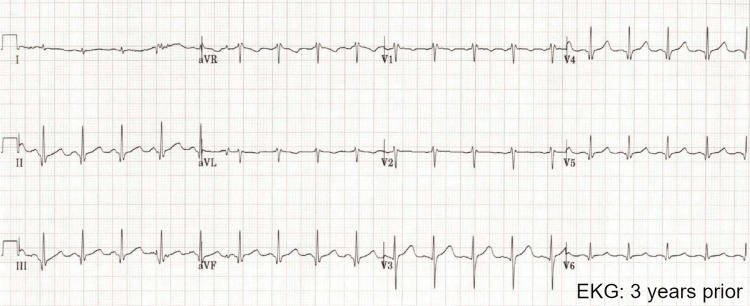
Patient EKG, three years prior to current admission, notable for normal sinus rhythm and incomplete right bundle branch block.

**Figure 2:**
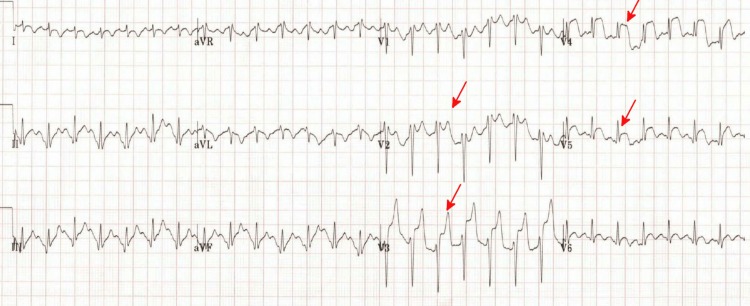
EKG on arrival to the emergency department. ST elevation is noted (red arrows) predominantly in the precordial leads (V2-V4), suggesting acute myocardial infarction.

While the patient’s prior hospital EKG (electrocardiogram) on file was unremarkable (Figure 1), the electrocardiogram (EKG) on arrival to the emergency department showed multiple abnormalities, including ST elevation in multiple leads (Figure 2). An initial discussion was held with the interventionalist on call, but due to a lack of antecedent symptoms consistent with myocardial infarction, clinical instability, and a high suspicion that an alternative diagnosis other than acute coronary syndrome was the primary issue, no invasive procedures were planned.

**Figure 3:**
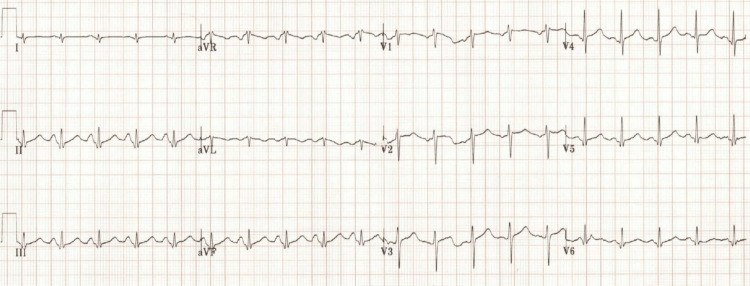
EKG performed approximately 24 hours after aggressive treatment of the patient with fluids, cooling, and supportive care. ST elevation has normalized.

The patient was initially treated with rapid administration of 4 l of cooled intravenous fluids, and his temperature improved to 100.8°F. Initial laboratory workup was significant for leukocytosis and acute kidney injury. He was started on broad-spectrum antibiotics, vancomycin and Zosyn, for suspected sepsis and admitted to the intensive care unit for further management. Repeat EKG done approximately 24 hours after the patient's initial presentation demonstrated complete normalization of the ST-segment elevation (Figure 3).

Over a protracted hospital course (lasting nearly six weeks), the patient was treated for shock, as well as disseminated intravascular coagulation (DIC), which required transfusion of platelets, packed red blood cells, fresh frozen plasma, and cryoglobulin. The patient developed acute kidney injury, was anuric, and required renal replacement therapy for approximately two weeks, but ultimately had complete renal function recovery. There was the further complication of invasive candidemia, requiring extended anti-fungal therapy. Consistent with the acuity of his illness, multiple laboratory abnormalities (Table 1) were noted, which normalized over the course of his hospital stay.

**Table 1:** Pertinent laboratory values on arrival, at peak abnormal level, and normalized value prior to patient discharge. Peak abnormality is listed as "same" if the initial value was the value of maximum derangement. eGFR: estimated glomerular filtration rate, AST: aspartate aminotransferase, ALT: alanine aminotransferase.

Parameter	Reference	Initial	Peak Abnormality	Normalized (Last) Value
Troponin	<22 ng/L	607	1,296	1,109
White blood cell	4.5-11.0 K/µL	25.8	Same	9.2
Hemoglobin	13.5-18.0 g/dL	18.5	Same	10.2
Platelets	140-440 K/µL	74	19	332
Lactate	0.5-2.0 mmol/L	7.66	Same	1.8
Creatinine	0.67-1.17 mg/dL	2.87	6.84	0.93
eGFR	≥60 mL/min/1.73 m^2^	28	10	118
AST	0-40 U/L	92	5,788	17
ALT	0-41 U/L	44	6,055	18

## Discussion

Malignant hyperthermia (MH) is a rare but life-threatening condition characterized by severe hyperthermia, muscle rigidity, and metabolic acidosis. It is commonly associated with the use of certain anesthetics and muscle relaxants, but other triggers, such as exercise and heat stroke, have been reported. Recently, an increasing number of cases of drug-induced hyperthermia have been reported, with methamphetamine use being a notable cause.

Methamphetamine stimulates the central nervous system, causing hyperthermia, tachycardia, and hypertension, and can lead to multi-organ failure and death. The mechanisms underlying drug-induced hyperthermia are not fully understood, but it is thought to be due to excessive activation of the sympathetic nervous system and/or direct toxic effects on the muscle cells [1]. Methamphetamine has been associated with a variety of cardiovascular effects, including hypertension, tachycardia, and arrhythmias. Methamphetamine increases sympathetic nervous system activity, leading to increased cardiac output and myocardial oxygen demand. It also induces vasoconstriction, impairing coronary blood flow and causing myocardial ischemia. Methamphetamine can cause direct cardiotoxicity as well, by inducing oxidative stress and mitochondrial dysfunction, which can lead to apoptosis and fibrosis of cardiac myocytes. Additionally, methamphetamine abuse is often accompanied by other unhealthy lifestyle choices such as poor nutrition, tobacco use, and lack of exercise, which can exacerbate cardiovascular disease risk factors [2].

The cornerstone of MH management is early recognition and discontinuation of the offending agent, typically anesthetic gases or depolarizing muscle relaxants. The administration of dantrolene, a skeletal muscle relaxant, is considered the gold standard of treatment for MH. Dantrolene works by inhibiting the release of calcium ions from the sarcoplasmic reticulum, which prevents excessive muscle contraction that leads to hyperthermia and metabolic acidosis. In addition to dantrolene, supportive measures such as cooling the patient and correcting metabolic acidosis are essential. Aggressive fluid resuscitation and vasopressor support may be required to maintain adequate blood pressure and tissue perfusion. In severe cases, renal replacement therapy and extracorporeal membrane oxygenation (ECMO) may be necessary [3].

ST elevation on an electrocardiogram (EKG) is a common finding in patients with acute myocardial infarction (AMI), but it can also be seen in a variety of other conditions. ST elevation can be caused by abnormalities in repolarization, either due to ischemia or injury, or by conditions that affect the balance of ions across the cell membrane. Other causes of ST elevation include pericarditis, early repolarization syndrome, myocarditis, and left ventricular hypertrophy. Medications, such as digitalis and quinidine, can also cause ST elevation. Additionally, hyperkalemia, hypothermia, and left bundle branch block can also cause ST elevation [4]. More uncommon causes can be seen in conditions such as Brugada syndrome, which can be unmasked during periods of high temperature or fever [5]. There is scarce literature regarding hyperthermia as a cause of ST elevation [6] or the link between this entity and methamphetamines [7]. The typical cause of ST elevation in the setting of methamphetamine includes plaque rupture, coronary dissection, and coronary spasm. Any of these can occur in the setting of existing plaque, which is itself promulgated by the chronic use of methamphetamines [8,9].

## Conclusions

In conclusion, malignant hyperthermia due to methamphetamine toxicity is a rare but potentially lethal condition that requires early recognition and prompt treatment. While the management of MH due to methamphetamine is similar to therapy for other causes, it is crucial to discontinue methamphetamine use in addition to other supportive measures. Despite the availability of treatment, the mortality rate associated with MH remains high, with mortality >50% in patients with very high core temperatures. Healthcare providers should maintain a high index of suspicion for MH in patients with methamphetamine toxicity and consider it as a differential diagnosis in those presenting with hyperthermia, altered mental status, and EKG changes. Early intervention and aggressive treatment remain keys to improving patient outcomes and reducing mortality, the high rate associated with this life-threatening condition.

## Figures and Tables

**Figure 4:**
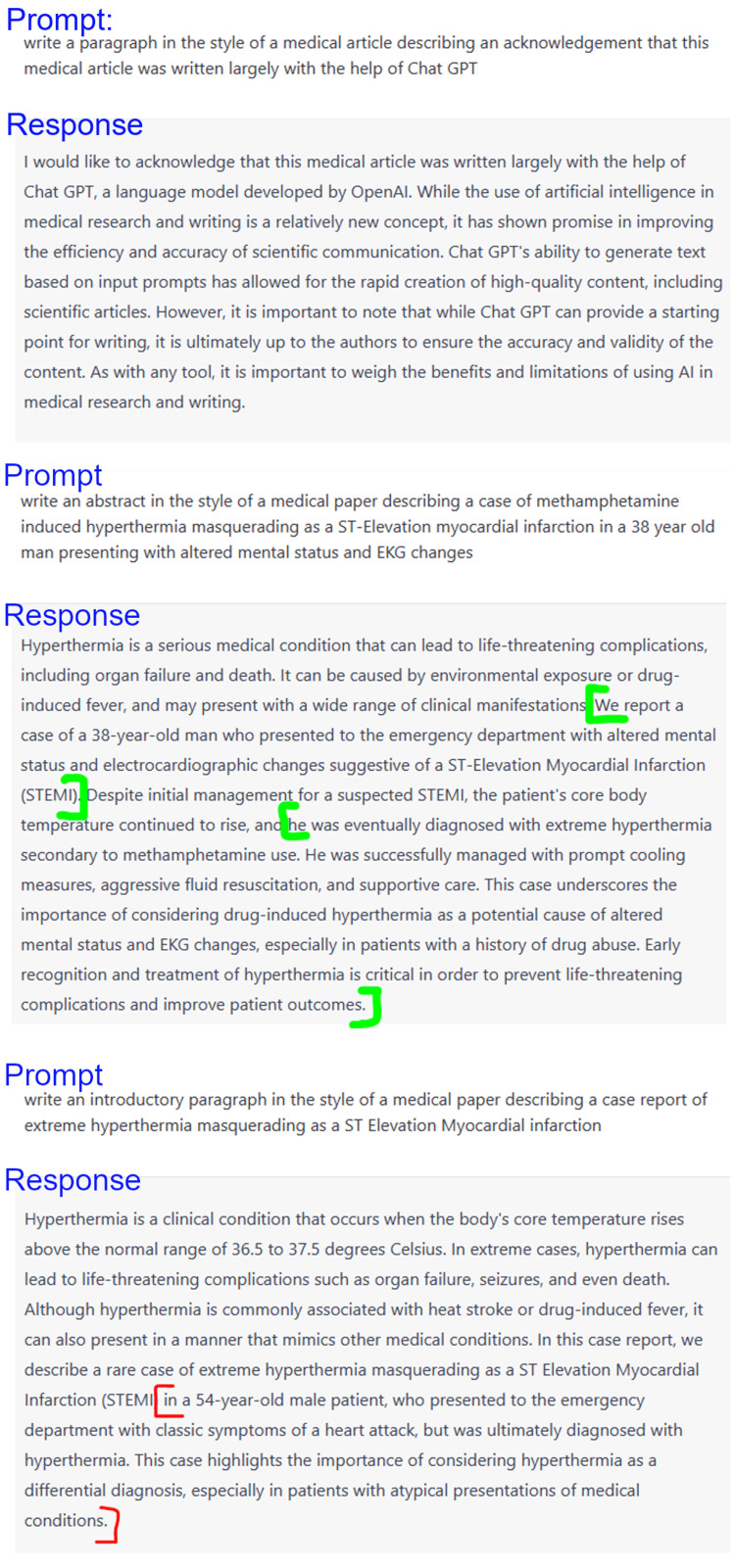
Examples of prompts given to ChatGPT and the responses that followed. In general, responses were highly cogent, well organized, and content was relevant to the prompt that was given. Responses were then used for portions of the paper, with minor revisions, or sections (green brackets) used. It is noted that entire portions of the responses could be completely erroneous (red brackets) if ChatGPT was not given explicit information as to the content request, and so careful attention had to be paid before the relevant portions were used for the paper.

## Appendices

ChatGPT was used extensively in the writing of this paper, as an aid in creating different sections before editing and submission. This is the first time the authors have used such a technique. Given the potential uses (and misuses) that such a tool can create, it bears further discussion of the exact methods whereby this was accomplished.

The process began by providing prompts to the model, based on an outline and references, which were then used to generate initial drafts of the various sections of the article. These sections were then revised and refined by the authors to ensure accuracy and clarity (Figure 4). While the use of ChatGPT enabled a more efficient drafting process, it was important to carefully review and edit the generated text to ensure its relevance, accuracy, and appropriateness.

The authors found the Artificial Intelligence (AI) very facile in creating cogent and grammatically well written constructs, which are very difficult to differentiate from content written by a human author. It bears emphasizing that while the output from this AI appears cogent and factual, when not given specific instructions, the AI tends to embellish and create factually inaccurate (albeit perfectly legitimate sounding) information. The second paragraph in this appendix was written entirely by ChatGPT, without the need for any editing. The first and third paragraphs were written by the human authors.
